# CD36 is essential for endurance improvement, changes in whole‐body metabolism, and efficient PPAR‐related transcriptional responses in the muscle with exercise training

**DOI:** 10.14814/phy2.13282

**Published:** 2017-05-19

**Authors:** Mark Christian C. Manio, Shigenobu Matsumura, Daisaku Masuda, Kazuo Inoue

**Affiliations:** ^1^ Laboratory of Nutrition Chemistry Division of Food Science and Biotechnology Graduate School of Agriculture Kyoto University Kyoto Japan; ^2^ Department of Cardiovascular Medicine Osaka University Graduate School of Medicine Osaka Japan

**Keywords:** CD36, endurance, PPAR activation, training adaptation, whole‐body metabolism

## Abstract

Although circulating fatty acids are utilized as energy substrates, they also function as ligands to the peroxisome‐proliferator activated receptors (PPARs), a family of fatty acid sensing transcription factors. Exercise training leads to various adaptations in the muscle such as elevation of glycogen content, mitochondrial number as well as upregulation of fatty acid uptake and utilization through downstream transcriptional adaptations. In line with this, CD36 has been shown to be critical in controlling fatty acid uptake and consequently, fatty acid oxidation. We show that exercise training could not ameliorate impaired endurance performance in CD36 KO mice despite intact adaptations in muscle glycogen storage and mitochondrial function. Changes in whole‐body metabolism at rest and during exercise were also suppressed in these animals. Furthermore, there was inefficient upregulation of PPAR and PPAR‐related exercise‐responsive genes with chronic training in CD36 KO mice despite normal upregulation of *Pgc1a* and mitochondrial genes. Our findings supplement previous observations and emphasize the importance of CD36 in endurance performance, energy production and efficient downstream transcriptional regulation by PPARs.

## Introduction

Fatty acids provide energy during metabolic challenges such as fasting and exercise. Uptake of fatty acids in cells occurs primarily by active transport facilitated by several fatty acid transporters such as fatty acid transport proteins (FATPs), fatty acid binding proteins (FABPs), and fatty acid translocase CD36 on the cell membrane which exhibit ligand binding preference [for review see Glatz et al. (2010)]. Expression of these proteins varies by cell type and their significance in organ function and whole‐body physiology are still being elucidated to date.

CD36 regulates different physiological functions in many metabolic organs such as the heart, adipose tissue and muscle as well as in nonmetabolic tissues such as nasal mucosa and these functions range from inflammation, nutrient absorption, metabolism, fatty acid sensing, and signal transduction with various endogenous ligands (Silverstein and Febbraio 2009; Lee et al. 2015). In the skeletal muscle, CD36 is the predominant fatty acid transporter that regulates fatty acid transport and consequently fatty acid oxidation (Nickerson et al. 2009; McFarlan et al. 2012) as this transporter localizes to the plasma membrane and mitochondrial outer membrane under different metabolic conditions (Luiken et al. 2002; Holloway et al. 2009; Jeppesen et al. 2011; Smith et al. 2011). As such, it is likely that this transporter also influences fatty acid‐mediated signaling in the organs it is expressed on.

Regular exercise training is an efficient stimulus for metabolic remodeling of the muscle [for review see Egan and Zierath (2013)]. Increased fatty acid catabolism, glycogen storage, shifting of substrate utilization from glucose to fat, oxidation of ketone bodies, muscle fiber transition to an oxidative phenotype and mitochondrial biogenesis and function have been observed in exercise training (Egan and Zierath 2013). These may be considered as adaptation mechanisms in the muscle to sustain energy production possibly to conserve glucose reserves for the brain (Nybo 2003) with consistent elevated energy demand. Transcription factors responsible for these changes in the muscle are the members of the peroxisome‐proliferator activated receptor (PPAR) family particularly PPAR*α* and PPAR*β*/*δ* as well as the estrogen‐related receptor (ERR) family (Huss et al. 2004; Schreiber et al. 2004; Wang et al. 2004; Hondares et al. 2007; Rangwala et al. 2010; Narkar et al. 2011; Gan et al. 2013). Common to these transcription factors is their activation by PPAR*γ*‐coactivator 1 *α* (PGC1*α*), which is upregulated and highly responsive to both acute and chronic exercise (Pilegaard et al. 2003; Lira et al. 2010). Although it has been established that fatty acids are ligands of PPARs and exercise training induces PPAR targets in the muscle, the role of CD36 in transcriptional adaptations with exercise has yet to be determined.

By employing whole‐body CD36 knockout mice, the role of CD36 in untrained mice on exercise performance and substrate utilization has been demonstrated (McFarlan et al. 2012; Fujitani et al. 2014). Exercise training in these mice upregulated mitochondrial proteins (McFarlan et al. 2012) but the consequence of this on endurance was not evaluated. In addition, several training‐induced adaptations in the muscle that could potentially compensate for the lack of CD36, that is to say impaired uptake and oxidation of fatty acids, during exhaustive exercise have yet to be investigated. In this study, we supplement these above reports and show that CD36 is essential not only for basal endurance performance but also for its training‐induced improvement. Also, we show that efficient transcriptional activation of exercise‐responsive genes in the muscle and downstream phenotypic manifestations in whole‐body metabolism are, in part, dependent on CD36.

## Materials and Methods

### Animal studies

Whole‐body CD36 knock‐out mice (KO) (hereby denoted as K) and wild‐type (WT) (hereby denoted as W) littermates were housed in a room maintained at 22 ± 0.5°C, 50% humidity and a 12 h light‐dark cycle (6:00 lights on; 18:00 lights off). These mice were received by our laboratory as a gift from Dr. Mason W. Freeman of Harvard Medical School (Moore et al. 2002) and was maintained on a C57BL6/J background. Mice were bred in‐house and from 4 week‐old mice were weaned, genotyped and had free access to a regular chow diet (Oriental Yeast Co., Tokyo, Japan) and water. Only male mice were used in this study. A purified diet containing 30% kcal from fat (soybean oil), 20% kcal from protein, and 50% kcal from carbohydrates (Research Diets, NJ) and water was provided ad libitum at 8 week‐old when experiments were commenced. Animal experiments as detailed in the following sections were conducted according to the Kyoto University Guidelines for the Ethical Treatment of Laboratory Animals as approved by the committee (No. 28‐28).

#### Exercise training

W and K at 8 week‐old were randomly assigned to either untrained (U) or exercised (E) groups and 30 days of training was commenced. On the first 15 days, mice ran at a constant speed of 15 m/min for 50 min on a motorized treadmill (MK‐680; Muromachi, Tokyo, Japan) kept an angle of 3°. On the last 15 day, time was increased to 75 min while other parameters were kept constant. Mice were stimulated to run by poking with a metal rod. All mice were responsive to stimulation and completed the training protocol. Training was done within the first 3 h of the light phase. Mice were subdivided into groups intended for basal indirect calorimetry, fixed‐time exercise, exercise‐to‐exhaustion test with indirect calorimetry, and exogenous glucose oxidation during exercise.

#### Whole‐body metabolism at rest

Basal indirect calorimetry was performed according to Manio et al. (2016). Mice were placed individually in acrylic chambers from the 3rd day before the last training session. Mice were left undisturbed for 48 h after the last training session. Mice had ad libitum access to food and water while in the chambers. Measurement was commenced from the acclimatization period with sampling every 10 min. However, only data from the final 24 h corresponding to a full light and dark cycle were analyzed to represent the chronic effect of training on basal whole‐body metabolism. Mice were sacrificed after measurements and time between the first and last mouse was kept within 2 h to avoid the effects of time differences in metabolite concentration and gene expression.

#### Fixed‐time exercise

Fixed‐time exercise was performed 48 h after the last training session. Mice were placed on a moving treadmill set at 10 m/min. After 2 min of warm‐up, mice were immediately transferred to a moving treadmill set at 15 m/min at an inclination of 10°. Mice were made to run for 1 h with an electrical stimulus of 0.2 mA. Mice were sacrificed after the run.

#### Whole‐body metabolism during exercise and exercise‐to‐exhaustion

Exercise‐to‐exhaustion test with indirect calorimetry was performed 48 h after the last training session. Mice were individually placed in airtight treadmill at an incline of 10° (Mousebelt‐200; Arco System, Tokyo, Japan) and were left undisturbed for 1.5 h. After a short warm‐up, mice were made to run for 3 h at 15 m/min followed by 1 h at 17 m/min. Intensity was increased to 19 m/min and kept herein until mice reached exhaustion. Indirect calorimetry was commenced upon assignment of mice to the treadmill. Similar measurement parameters were employed as in the basal indirect calorimetry and only the time interval of sampling was changed to 2 min. Mice were forced to run by mild electrical stimulus of 0.2 mA. At the point of exhaustion, mice were immediately sacrificed. Mice were declared exhausted after failing to respond or sustain running for 20 sec despite poking and tapping on chamber walls with occasional elevation of electrical stimulus. Exhaustion was confirmed by serum glucose, muscle, and liver glycogen depletion (not shown).

#### Single bout of exercise

W and K at 8‐week‐old were fed the same purified diet for 32 day. At the onset of the light phase, mice were assigned into no run (N) or 1× run (R) groups and made to run for 50 min at 15 m/min, 3° incline similar to the training protocol. Mice were returned to cages and allowed to recover normally with access to food and water. Mice were sacrificed after 8 h.

#### Exogenous glucose oxidation during exercise

Exogenous glucose oxidation using ^13^C‐labeled glucose was based on Fujitani et al. (2014). Forty‐eight (48) h after the last training session, mice were placed individually in treadmill chambers for indirect calorimetry. One hour after acclimatization, ^13^C‐labeled d‐glucose (Cambridge Isotope Laboratories, Inc., MA) in water was administered orally at a dose of 36 mg/kg BW (10 mL/kg BW) and mice were returned to chambers. After 8 min, the run was commenced starting with a brief warm‐up at 10 m/min then increased to 15 m/min for a total of 30 min. Expired CO_2_ expressed as %^13^C/^12^C as calculated by the accompanying software was plotted against time.

### Sample collection

Mice were sacrificed by decapitation. Blood was collected and an aliquot was deproteinized by immediately mixing (1:1) with 0.8 mol/L perchloric acid. After 5 min, the mixture was centrifuged at 1.5 × 1000 *g* for 5 min at 4°C. The resulting supernatant was aliquoted and flash frozen in liquid nitrogen. Remaining whole blood was left to coagulate for 30 min to 1 h and serum was collected after centrifugation at 1.5 × 1000 *g* for 10 min at 4°C. Gastrocnemius and liver samples were clamp frozen in liquid nitrogen. All samples were transferred to a −70°C freezer until processing. Clamp frozen gastrocnemius and liver were powdered using a liquid nitrogen‐cooled mortar and pestle, aliquoted and weighted for glycogen, lipid, protein, total RNA, and acid‐soluble compounds extraction. Samples were returned to the same storage conditions.

### Blood chemistry

Kits were used to analyze blood chemistry. Serum was analyzed for glucose, nonesterified fatty acids (NEFA), triglycerides (TG), and *β*‐hydroxybutyrate (*β*‐HB) (Wako, Osaka, Japan) while deproteinized blood was analyzed for lactate (Kyowa Medex, Tokyo, Japan) according to manufacturers’ instructions.

### Glycogen

Glycogen measurement was based on Good et al. (1933) and Sahyun and Alsberg (1931) with some modifications. In brief, approximately 25 mg powdered muscle and liver samples were digested in alkali with 300 *μ*L 30% KOH at 100 °C for 30 min. Precipitation of glycogen was carried out by addition of 50 *μ*L saturated Na_2_SO_4_ and 500 *μ*L cold ethanol followed by centrifugation at room temperature (RT) for 2.3 × 1000 *g* for 5 min. The pellet was washed with 500 *μ*L distilled water, vortexed and precipitated with 625 *μ*L ethanol followed by centrifugation. The pellet was dehydrated to completeness at 50–70°C for 1.5–2 h. The pellet was suspended in 500 *μ*L 0.6 mol/L HCl. An aliquot was digested for 3 h at 99°C using a thermal cycler with a heated lid to prevent evaporation. Liberated glucose was measured similar to serum glucose and weight of glycogen was calculated from this value using a formula that assumes all glucose units are linearly polymerized into one molecule of glycogen.

### Muscle lipids

Lipids were extracted from powdered muscle samples as described in Manio et al. (2016) with some modifications. Approximately 30 mg samples were mixed thoroughly with 1 mL Folch reagent. After more than 16 h incubation at 4°C in a revolving mixer, 200 *μ*L of 4 mmol/L MgCl_2_ was added and vortexed. Following 10 min incubation at 4°C, samples were centrifuged at 1.2 × 1000 *g* for 1 h. From the bottom chloroform layer, 500 *μ*L was collected and evaporated to dryness at 40°C for about 2 h. The desolvated lipids from muscle samples were suspended in 50 *μ*L of 10% Triton‐X in isopropanol. Measurement of TG and NEFA was similar to serum samples.

### Muscle lactate and *β*‐HB

Lactate and *β*‐HB were extracted from muscle samples based on Ohtsu et al. (2003) with some modifications. In brief tissues were incubated in 3 volumes of 1 mol/L PCA with occasional shaking for 1 h at 4°C. After centrifugation at 10 × 1000 *g* for 2 min, the supernatant was collected. For every 50 *μ*L of PCA extract, 21.5 *μ*L of 1 mol/L K_2_CO_3_ was added to neutralize the solution between pH 6.5–7.5. The precipitate was spun down and the supernatant was aliquoted and stored at −70°C until analysis. Measurement of lactate and *β*‐HB was similar to blood chemistry samples.

### Real‐time quantitative polymerase chain reaction (RT‐qPCR)

Total RNA was extracted from approximately 20 mg powdered gastrocnemius samples with Tripure Isolation Reagent (Roche, Mannheim, Germany) followed by purification using Genelute Mammalian Total RNA Miniprep Kit (Sigma‐Aldrich, MO). In brief, frozen samples were sonicated in the isolation reagent to ensure complete lysis followed by addition of chloroform according to the reagent's protocol. After centrifugation, supernatant was transferred to the blue column of the kit to shred potential protein and DNA contaminants. From here, the procedure was according to the manufacturer's instructions. Total RNA (1.8 *μ*g) was reverse transcribed with Transcriptor First Strand cDNA Synthesis Kit (Roche, Mannheim, Germany) according to manufacturer's instructions. Messenger RNA (mRNA) levels were amplified with Premix ExTaq for probe qPCR (Takara Bio, Shiga, Japan) using primers that span intronic regions for mRNA specificity and corresponding Universal Library Probes (Roche, Mannheim, Germany) as listed in Table 1. Values were rationalized to *Hprt* expression (Cappelli et al. 2008).

**Table 1:** RT‐qPCR primers and probes

mRNA primers		Sequence (5′ to 3′)	Universal Probe No.	Accession no.
Pgc1a	Forward	tgtggaactctctggaactgc	63	NM_008904.2
	Reverse	agggttatcttggttggcttta		
Ppara	Forward	ccgagggctctgtcatca	11	NM_011144.6
	Reverse	gggcagctgactgaggaa		
Ppard	Forward	atgggggaccagaacacac	11	NM_011145.3
	Reverse	ggaggaattctgggagaggt		
Cycs	Forward	aacgttcgtggtgttgacc	104	NM_007808.4
	Reverse	ttatgcttgcctcccttttc		
Pdk4	Forward	cgcttagtgaacactccttcg	22	NM_013743.2
	Reverse	cttctgggctcttctcatgg		
Oxct1	Forward	caggcaatgtgattttcagg	22	NM_024188.6
	Reverse	gcaaatgagccaatgtctacaa		
Cd36	Forward	tttcctctgacatttgcaggt	11	NM_001159558.1
	Reverse	gattctggaggggtgatgc		
Fatp1	Forward	cttcctaaggctgccattgt	49	NM_011977.3
	Reverse	ggcagtcatagagcacatcg		
Cpt1b	Forward	ccatcattgggcacctct	104	NM_009948.2
	Reverse	gtctccgtgtagcccaggt		
Lpl	Forward	tggataagcgactcctacttcag	22	NM_008509.2
	Reverse	tccctagcacagaagatgacc		
Myh7	Forward	tgcattgacctcatcgagaa	63	NM_080728.2
	Reverse	gtcatgtctgtggccttgg		
Myh4	Forward	tgcttacgtcagtcaaggtga	104	NM_010855.2
	Reverse	aatcccaggatatcaacagca		
Myh2	Forward	tcttctctggggcacaaact	22	NM_001039545.2
	Reverse	cccttcttcttggcaccttt		
Glut4	Forward	tcgtcattggcattctggt	104	NM_009204.2
	Reverse	agcagtggccacagggta		
Vegfa	Forward	actggaccctggctttactg	22	NM_001025250.3
	Reverse	tctgctctccttctgtcgtg		
Mct1	Forward	atgctgccctgtcctcct	49	NM_009196.3
	Reverse	ccacaagcccagtacgtgtat		
Mct4	Forward	ctcatagatctttatgactggacagg	11	NM_146136.1
	Reverse	tgtttgattggctgtggatg		
Hprt	Forward	cctcctcagaccgcttttt	95	NM_013556.2
	Reverse	aacctggttcatcatcgctaa		

### Protein extraction

Powdered muscle samples were lysed in a buffer containing 50 mmol/L Tris, 150 mmol/L NaCl, 1 mmol/L EDTA, 2 mmol/L MgCl_2_, 1 mmol/L mercaptoethanol and 1% NP‐40 adjusted to pH 8.0 and supplemented with protease and phosphatase inhibitor cocktails (Roche, Mannheim, Germany) according to manufacturer's instructions. Insoluble matter was precipitated by centrifugation at 10 × 1000 *g* for 20 min at 4°C. Supernatant was collected and transferred to a clean tube. The concentration was measured by Bradford protein assay and an aliquot was adjusted with lysis buffer to 2 *μ*g/*μ*L. This was further aliquoted for enzyme activity measurements and immunoblot experiments. The concentration of this adjusted aliquot was measured to assure accurate loading of samples in succeeding experiments. The original lysate and adjusted lysate were snap frozen in liquid nitrogen before storing at −70°C.

### Enzyme activities

Beta‐hydroxyacyl‐CoA dehydrogenase (*β*‐HAD) activity was measured based on Holloway et al. (2007) with some modifications. 4 *μ*L of adjusted lysate containing approximately 8 *μ*g protein was pipetted to a 96‐well plate. All solutions were warmed to 37°C prior to addition. To wells, 100 *μ*L of a solution containing 0.5 mmol/L NADH in 50 mmol/L Tris and 2 mmol/L EDTA at pH 7.0 was pipetted. The plate was briefly shaken followed by 5 min incubation at 37°C. After incubation, 100 *μ*L of a solution containing 0.2 mmol/L acetoacetyl‐CoA in the same buffer was pipetted, and the plate briefly shaken before reading at 340 nm for 10 min at 37°C on a kinetic program.

The forward reaction of succinyl‐CoA: 3‐oxoacid CoA‐transferase (SCOT) activity was measured based on Williamson et al. (1971) with some modifications. A general buffer containing 50 mmol/L Tris, 10 mmol/L MgCl_2_ and 5 mmol/L iodoacetamide at pH 8.5 was prepared. To an aliquot, 50 mmol/L sodium succinate was prepared. To another aliquot, 0.1 mmol/L acetoacetyl‐CoA was prepared. 4 *μ*L of adjusted lysate containing approximately 8 *μ*g protein was pipetted to a 96‐well plate. To wells, 190 *μ*L of acetoacetyl‐CoA solution was added and incubated for 5 min at RT followed by the addition of 10 *μ*L of succinate solution. The plate was briefly shaken before reading at 303 nm for 10 min at RT on a kinetic program.

The activity of pyruvate‐to‐lactate reaction of lactate dehydrogenase (LDH) was measured according to Crabtree and Newsholme (1972) and Howell et al. (1979) with some modifications. A reaction buffer containing 0.1 mmol/L pyruvate and 0.15 mmol/L NADH in 50 mmol/L potassium phosphate buffer at pH 7.4 was prepared and incubated at 37°C prior to reaction. To 5 *μ*L of adjusted lysate containing approximately 10 *μ*g protein, 200 *μ*L of the reaction buffer was pipetted, briefly shaken and read at 340 nmol/L for 10 min at 37°C on a kinetic program.

Citrate synthase (CS) activity was measured based on Manio et al. (2016) and Srere (1969). Protein concentration of the final adjusted lysate was used in all the calculations. In all enzyme activity assays, blank wells contained the lysis buffer.

### Immunoblotting

Muscle lysates were adjusted with lysis buffer and 4× Laemilli buffer to achieve a concentration of 1.25 *μ*g/*μ*L. 40 *μ*g of protein was subjected to electrophoresis in a 10% polyacrylamide gel. Transfer to PVDF membranes was performed in transfer buffer containing 20% methanol for 1 h. Membranes were incubated with shaking for 2 min in 0.5% Ponceau S stain. After three washings in distilled water for 1 min each with shaking, membranes were sandwiched in a plastic film, visualized and digitized (LAS‐3000; Fujifilm, Tokyo, Japan). Membranes were cut into two pre‐determined sections known to contain CD36 and PDK4 protein. Membranes were blocked in Block Ace Powder (DS Pharma Biomedical, Osaka, Japan) in Tris‐buffered saline with 0.1% Tween‐20 (TBS‐T) buffer according to manufacturer's instructions followed by incubation overnight at 4°C in anti‐CD36 primary antibody (1:2000; AF2519, R&D Systems, MN) or anti‐PDK4 primary antibody (1:1000; SC‐14495, Santa Cruz Biotechnology, CA) diluted in blocking solution. Membranes were washed in three replacements of 20 mL TBS‐T buffer for 5 min each with shaking before incubation in horseradish peroxidase‐labeled anti‐goat IgG secondary antibody (1:1000; P0449, Dako, Tokyo, Japan) for 3 h at 4°C. Membranes were washed with TBS‐T buffer thrice and reacted for chemiluminescence detection with Western Lightning Plus ECL (Perkin Elmer, MA) for 1 min. Detection and visualization using the LAS‐3000 system was performed every 10 sec for 3.5 and 8.5 min for CD36 and PDK4, respectively. Band intensity at 88 kDa and 47 kDa corresponding to CD36 and PDK4, respectively and Ponceau S stain signals were quantified using the software MultiGauge V3.2 (Fujifilm, Tokyo, Japan) with automatic background detection.

### Statistical analyses

Statistical analyses were performed on the software Prism 5.0 (Graphpad Software, CA). Indirect calorimetry time‐course data are presented as means. All other data are presented as mean ± SEM. To determine genotype and treatment interaction effect, and group x time differences in exogenous glucose oxidation, two‐way Analysis of Variance (ANOVA) was performed wherever appropriate. Between genotype (same treatment), between treatment (same genotype) and metabolite concentration (between pre‐ and post‐exercise) comparisons were performed using unpaired Student's *t*‐test at a significance level of *α *= 0.05.

## Results

**Figure 1:**
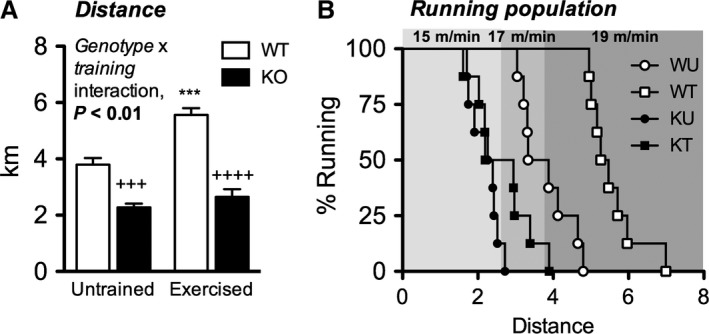
CD36 is necessary for basal endurance and its training‐induced improvement. (A) Distance covered until attaining exhaustion under a forced running exercise‐to‐exhaustion protocol and (B) percentage of mice running at a specific intensity and distance. Data in (A) are expressed as mean ± SEM (*n* = 7–8). Interaction effect was assessed by two‐way ANOVA. Between group differences were assessed by Student's unpaired *t*‐test where triple and quadruple symbols represent *P* < 0.001 and 0.0001, respectively. *represents significant difference between exercise status of the same genotype while ^+^represents significant difference between genotypes of the same exercise status. Each data point in (B) represents an individual mouse plotted against the distance covered by the said mouse at exhaustion.

To determine the effect of training on endurance, mice were subjected to exercise‐to‐exhaustion test. Training improved endurance in W (46.7%; *P *< 0.001). As previously reported (McFarlan et al. 2012; Fujitani et al. 2014), K were exercise intolerant relative to W counterparts (*P* < 0.001) (Fig. 1A and B). Despite completing the same training program, exercise intolerance in KU was not ameliorated in KE. Furthermore, KU and KE were not able to sustain running and most reached exhaustion before the increase in intensity to 17 m/min and 19 m/min in contrast to their W counterparts (Fig. 1B).

**Figure 2:**
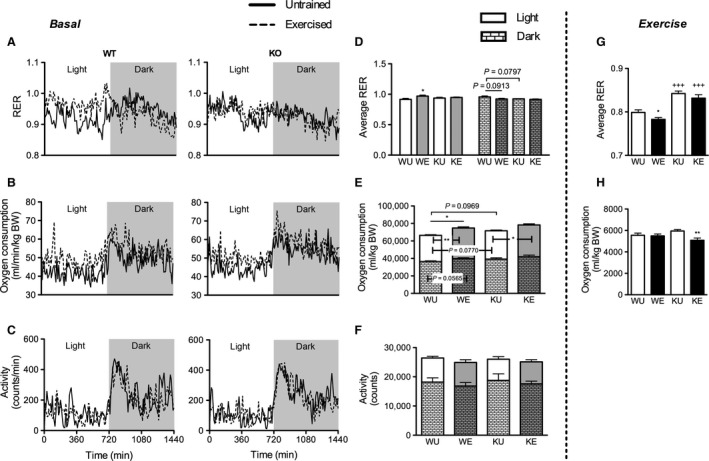
Changes in whole‐body metabolism at rest (basal) and during exercise. (A–C) Basal time‐course changes in RER, oxygen consumption, and activity of WT (left) and CD36 KO (right) mice for 24 h. (D–F) Corresponding average RER, and cumulative oxygen consumption and activity counts in total and relative contributions during light and dark phases. (G–H) Average RER and cumulative oxygen consumption of mice during a 60 min run. Each point in the time course data are expressed as means while average and cumulative data are expressed as mean ± SEM (*n* = 7–9). No significant interaction effect was observed as assessed by two‐way ANOVA. Between group differences were assessed by Student's unpaired *t*‐test where single, double and triple symbols represent *P* < 0.05, 0.01 and 0.001, respectively. *represents significant difference between exercise status of the same genotype while +represents significant difference between genotypes of the same exercise status. For basal whole‐body metabolism, annotations within the bars represent differences within the photophase while above the bars represent 24 h differences.

To assess basal whole‐body metabolism, mice were left undisturbed after the last bout of exercise for 48 h. Respiratory gases were measured during this period and data from the last 24 h was analyzed. The first 24 h was not used as it reflected whole‐body metabolism immediately after exercise toward recovery. In contrast to K, significant increase in average respiratory exchange ratio (RER) in the light phase in WE relative to WU (*P* < 0.05) and a nonsignificant decrease (*P = *0.0913) in the dark phase (Fig. 2A and D) were observed. Also, KU was lower (*P* = 0.0797) relative to WU in the dark phase. Total oxygen consumption was significantly increased (*P* < 0.05) in WE relative to WU which reflected the increases in both light and dark phases (*P* < 0.01 and *P* = 0.0565, respectively) (Fig. 2B and E). In K, oxygen consumption was significantly higher (*P* < 0.05) in KE relative to KU only in the light phase. These changes in whole‐body metabolism were not because of changes in spontaneous motor activity (Fig. 2C and F). Also, body weight was not different between genotypes and this was not affected by the training protocol (not shown). While no significant increase in food intake because of training was observed, both K groups had higher food intake relative to W counterparts (not shown).

Whole‐body metabolism during exercise was analyzed during the first 60 min where all mice were able to run without additional stimulation. Thus, this reflected whole‐body metabolism purely due to exercise and not because of external stimulation. K had higher RER (*P* < 0.001) than W counterparts (Fig. 2G). Lower RER (*P* < 0.05) was observed in WE relative to WU. In contrast, this decrease was not observed in KE relative to KU. Oxygen consumption was not affected in the W while lower values were observed in KE relative to KU (*P* < 0.01) (Fig. 2H).

**Figure 3:**
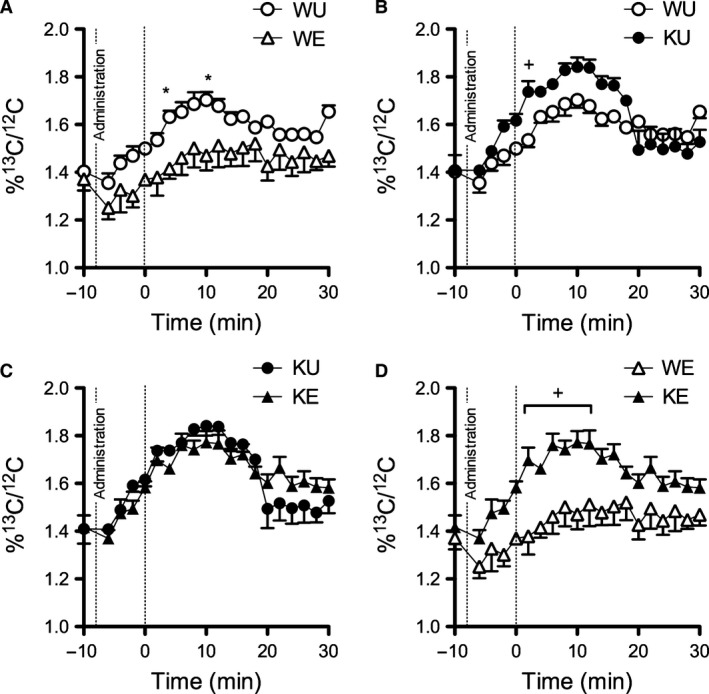
Training‐induced changes in whole‐body exogenous glucose oxidation is absent in CD36 KO mice. (A–D) Exogenous glucose oxidation using ^13^C‐labeled glucose in untrained and exercised mice during a 30 min run. Data are expressed as mean ± SEM (*n* = 5–7). Differences (*P* < 0.05) at certain time points were observed as assessed by two‐way ANOVA followed by Bonferroni's posthoc test. *represents significant difference between exercise status of the same genotype while +represents significant difference between genotypes of the same exercise status.

To assess exogenous glucose oxidation during exercise, ^13^C‐labeled glucose was administered to mice and running was commenced. Values of expired ^13^C‐labeled CO_2_ expressed as %^13^C/^12^C were significantly lower (*P* < 0.05) in WE relative to WU (Fig. 3A). Significant differences (*P* < 0.05) were observed between W and K in both training states (Fig. 3B, D) while significant difference was not observed between KU and KE (Fig. 3C). Significant differences in oxygen consumption were not observed between genotypes and training states during the test (not shown). No exercise effect was observed in glucose disposal by IPGTT after an 8 h fast and after an exercise bout although K had generally better glucose disposal than W counterparts (not shown).

Metabolites in the blood, muscle and liver at basal and postexercise states are summarized in Table 2. Basal serum glucose was lower (*P* < 0.05) in KE relative to KU. Postexercise glucose was higher in WU, WE, and KE (*P* < 0.0001–0.05) but not in KU. The increase in KE was not significantly different with KU. Both K groups had lower serum glucose than W counterparts postexercise (*P* < 0.0001 and *P* = 0.0507 for KU and KE, respectively). Basal muscle glycogen was higher in KU relative to WU (*P* < 0.05). Training increased muscle glycogen in WE and KE (*P* < 0.0001–0.05) but the degree of elevation in KE was 12% less (79.4% increase relative to KU) than WE (91.4% increase relative to WU). After exercise, muscle glycogen content significantly decreased and was similar in all groups (*P* < 0.0001). Basal blood lactate was not significantly different in all groups. WE and KE had lower blood lactate after exercise (*P* < 0.01 and *P* = 0.0593, respectively). Basal muscle lactate, on the other hand, was significantly higher in KE relative to KU and WE (*P* < 0.05). After exercise, no significant change was observed in WU and KU as well as KE that remained significantly elevated relative to KU. WE however, had significantly increased muscle lactate compared with basal values. Basal liver glycogen was lower in KU relative to WU (*P* < 0.001). Training decreased liver glycogen in WE (*P* < 0.01) while not significantly affecting KE. After exercise, significant decrease in WU and a nonsignificant decrease in KE (*P* < 0.001 and *P* = 0.0645, respectively) was observed. At exhaustion, muscle and liver glycogen were depleted and serum glucose decreased to similar levels in all groups (not shown).

**Table 2:** Blood, muscle, and liver metabolites at rest and immediately postexercise

Parameter	Time	Untrained		Exercised	
		WT	KO	WT	KO
Serum glucose (mg/dL)	Basal	144.69 ± 7.64	150.19 ± 9.64	156.50 ± 7.87	137.02 ± 2.88^a^
	1 h run	206.79 ± 7.49^c^	158.46 ± 5.61^b^	190.24 ± 12.29^c^	162.89 ± 3.57^c^
Muscle glycogen (mg/g)	Basal	1.269 ± 0.103	1.759 ± 0.185^b^	2.382 ± 0.099^a^	2.395 ± 0.164^a^
	1 h run	0.469 ± 0.073^c^	0.544 ± 0.112^c^	0.320 ± 0.039^c^	0.335 ± 0.067^c^
Liver glycogen (mg/g)	Basal	57.91 ± 3.10	31.38 ± 5.30^b^	39.50 ± 4.47^a^	43.75 ± 5.07
	1 h run	32.01 ± 4.54^c^	23.67 ± 3.70	30.44 ± 5.58	30.79 ± 3.84
Blood lactate (mg/dL)	Basal	21.87 ± 1.60	20.61 ± 1.72	23.50 ± 1.01	21.29 ± 0.91
	1 h run	19.40 ± 1.28	17.60 ± 1.01	18.79 ± 0.80^c^	18.24 ± 1.20
Muscle lactate (mg/g)	Basal	1.276 ± 0.100	1.067 ± 0.066	1.133 ± 0.060	1.373 ± 0.086^a^^,^ b
	1 h run	1.349 ± 0.050	1.161 ± 0.087	1.435 ± 0.045^c^	1.555 ± 0.090^a^
Serum NEFA (mEq/L)	Basal	0.694 ± 0.049	0.913 ± 0.050^b^	0.426 ± 0.032^a^	0.814 ± 0.037^b^
	1 h run	0.787 ± 0.028	1.031 ± 0.052^b^	0.762 ± 0.039^c^	1.009 ± 0.035^b^^,^ c
Serum TG (mg/dL)	Basal	101.28 ± 15.87	65.50 ± 5.39	73.00 ± 11.09	60.22 ± 5.04
	1 h run	105.08 ± 13.56	52.17 ± 2.38^b^^,^ c	91.13 ± 11.24	58.78 ± 3.54^b^
Serum *β*‐HB (*μ*M)	Basal	54.50 ± 9.57	75.07 ± 13.77	46.93 ± 4.20	71.17 ± 15.07
	1 h run	274.38 ± 37.09^c^	638.96 ± 60.24^b^^,^ c	250.11 ± 28.88^c^	503.22 ± 52.97^b^^,^ c
Muscle NEFA (mEq/g)	Basal	0.0127 ± 0.0011	0.0157 ± 0.0018	0.0097 ± 0.0010	0.0094 ± 0.0009^a^
	1 h run	0.0039 ± 0.0005^c^	0.0040 ± 0.0003^c^	0.0042 ± 0.0002^c^	0.0049 ± 0.0003^c^
Muscle TG (mg/g)	Basal	10.918 ± 1.552	12.627 ± 1.564	7.911 ± 0.926	7.290 ± 1.809^a^
	1 h run	1.876 ± 0.459^c^	1.510 ± 0.325^c^	1.732 ± 0.147^c^	1.225 ± 0.108^b^^,^ c
Muscle *β*‐HB (*μ*mol/g)	Basal	0.1027 ± 0.0341	0.1452 ± 0.0506	0.0882 ± 0.0093	0.1638 ± 0.0542
	1 h run	0.0691 ± 0.0109	0.1611 ± 0.0164^b^	0.0620 ± 0.0073^c^	0.1457 ± 0.0146^b^

Metabolites in the blood, muscle, and liver were measured at the basal state and after 1 h exercise. Data are expressed as mean ± SEM (*n* = 7–9). No significant interaction effect was observed as assessed by two‐way ANOVA. Between group differences were assessed by Student's unpaired *t*‐test where symbols represent *P* < 0.05–0.001.

a Represents significant difference between exercise status of the same genotype.

b Represents significant difference between genotypes of the same exercise status.

c Represents significant difference between basal and 1 h run data.

Lipid metabolites in the blood and muscle were also measured. Basal and postexercise serum NEFA was higher (*P* < 0.0001–0.01) in the K groups relative to W counterparts. Training lowered basal serum NEFA in WE (*P* < 0.001) but not in KE. After exercise, WE and KE had significantly higher serum NEFA relative to basal level (*P* < 0.0001 and *P* < 0.001, respectively) with WE attaining similar values as WU. Basal serum TG was lower but not significant in untrained groups relative to trained counterparts in both W and K. KU had significantly lower serum TG from basal (*P* < 0.05) albeit a small change after exercise. No change was observed in the other groups compared with basal values. However, at this period, serum TG was significantly lower in K groups relative to W counterparts (*P* < 0.01–0.05). Basal serum *β*‐HB was not significantly different between groups. After exercise, all groups had significant increases relative to basal values (*P* < 0.0001) and values in K were significantly higher relative to W counterparts (*P* < 0.0001–0.001). Basal intramuscular NEFA was lower in WE and KE relative to untrained counterparts (*P* = 0.0679 and *P* < 0.01, respectively). After exercise, values significantly decreased in all groups (*P* < 0.0001–0.001). Basal muscle TG was lower in trained groups but significantly different only between KE and KU (*P* < 0.05). After exercise, all groups had decreased muscle TG (*P* < 0.0001–0.01). KE, however, was significantly lower than WE (*P* < 0.05). Basal intramuscular *β*‐HB appeared to be higher but not significantly different in K groups relative to W counterparts. After exercise, a small but significant decrease from basal value was observed in WE (*P* < 0.05) but not in other groups. K at this state was significantly higher than W counterparts (*P* < 0.0001–0.001).

**Figure 4:**
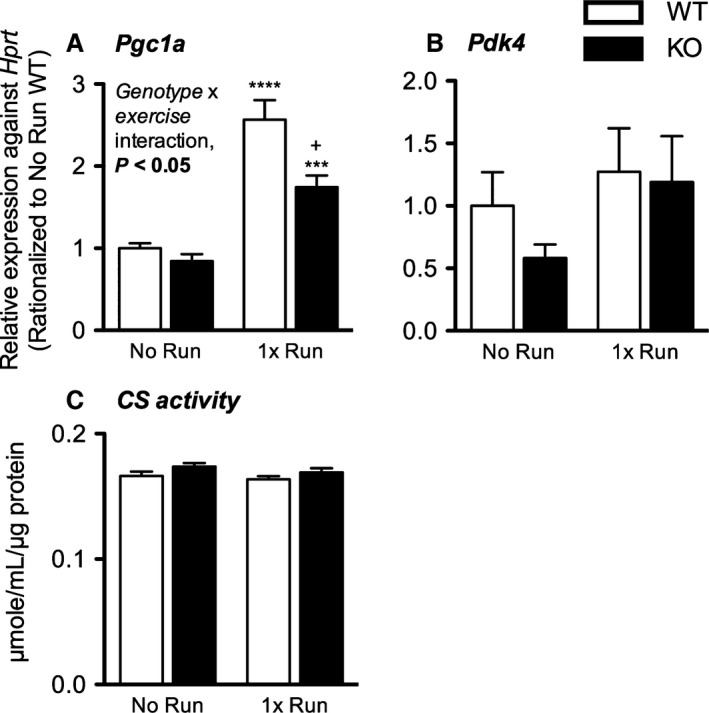
CD36 is required for efficient transcriptional response of *Pgc1a* during recovery after a single bout of exercise. (A) *Pgc1a* and (B) *Pdk4* mRNA expression, and (C) CS activity in gastrocnemius of WT and CD36 KO mice 8 h after a single bout of treadmill running. Data are expressed as mean ± SEM (*n* = 7–10). Interaction effect was assessed by two‐way ANOVA. Between group differences were assessed by Student's unpaired *t*‐test where single, triple, and quadruple symbols represent *P* < 0.05, 0.001 and 0.0001, respectively. *represents significant difference between exercise status of the same genotype while +represents significant difference between genotypes of the same exercise status.

Sedentary W and K mice ran on a treadmill to observe the influence of CD36 on early response gene transcription after a single bout of exercise. 8 h during recovery, *Pgc1a* but not *Pdk4* mRNA expression was significantly upregulated in the muscle of WR and KR relative to WN and KN, respectively (*P* < 0.0001–0.001) (Fig. 4A, B). Interestingly, the increase in transcription of this coactivator was significantly higher in WR relative to KR (*P* < 0.05; interaction effect, *P* < 0.05). CS activity did not change with a single bout of exercise in both genotypes (Fig. 4C).

**Figure 5:**
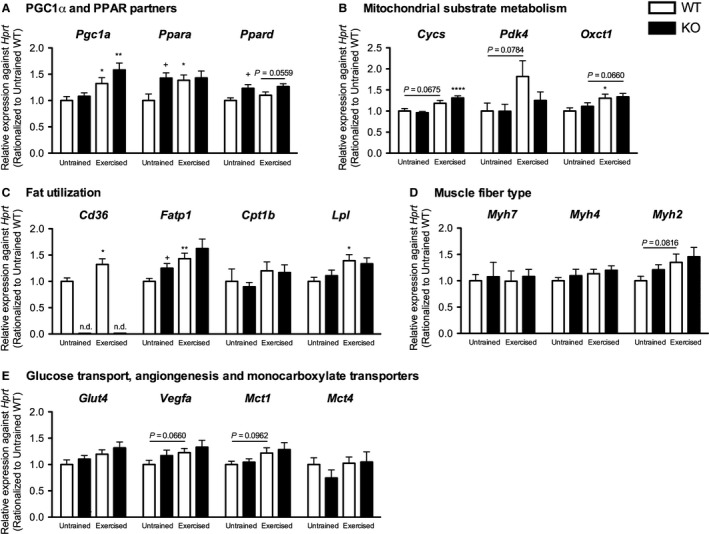
Changes in messenger RNA expression after training in WT and CD36 KO mice. Messenger RNA expression levels of (A) PGC1a and peroxisome‐proliferator activated receptors, and genes related to (B) mitochondrial substrate metabolism, (C) fat utilization, (D) muscle fiber type and (E) glucose transport, angiogenesis and monocarboxylate transporters in the gastrocnemius of WT and CD36 KO mice after 30 day of treadmill training. Data are expressed as mean ± SEM (*n* = 7–9). No significant interaction effect was observed as assessed by two‐way ANOVA. Between group differences were assessed by Student's unpaired *t*‐test where single, and double symbols represent *P* < 0.05, and 0.01, respectively. *represents significant difference between exercise status of the same genotype while +represents significant difference between genotypes of the same exercise status.

We determined the influence of CD36 on mRNA expression of exercise adaptation genes after training. *Pgc1a* was significantly increased with training in both genotypes (*P* < 0.01–05) (Fig. 5A). No difference between genotypes was observed in contrast to adaptation after a single bout of exercise (Fig. 4A). Transcription factors *Ppara* and *Ppard* were not influenced by training in KE while *Ppara* but not *Ppard* was significantly upregulated (*P* < 0.05) in WE (Fig. 5A). Interestingly, levels of both transcription factors were significantly higher in KU than WU (*P* < 0.05). In WE, nonsignificant increases in *Cycs* and *Pdk4* and a significant increase (*P* < 0.05) in *Oxct1* was observed relative to WU. While in KE, significant increase in *Cycs* (*P* < 0.0001) and a nonsignificant increase in *Oxct1* was observed relative to KU (Fig. 5B) suggesting increased mitochondrial gene transcription.

Fat utilization genes (*Cd36*,* Fatp1*, and *Lpl*) were significantly upregulated by training in WE (*P* < 0.01–0.05) (Fig. 5C). *Lpl* and *Fatp1* were also increased with training in KE but these were not significantly different with KU. Intriguingly, *Fatp1* was significantly higher in KU relative to WU (*P* < 0.05). Nonsignificant increases were observed in the gene corresponding to myosin heavy chain type IIb (*Myh4*), *Vegfa* and *Mct1* in trained groups relative to untrained counterparts of both genotypes (Fig. 5D, E).

**Figure 6:**
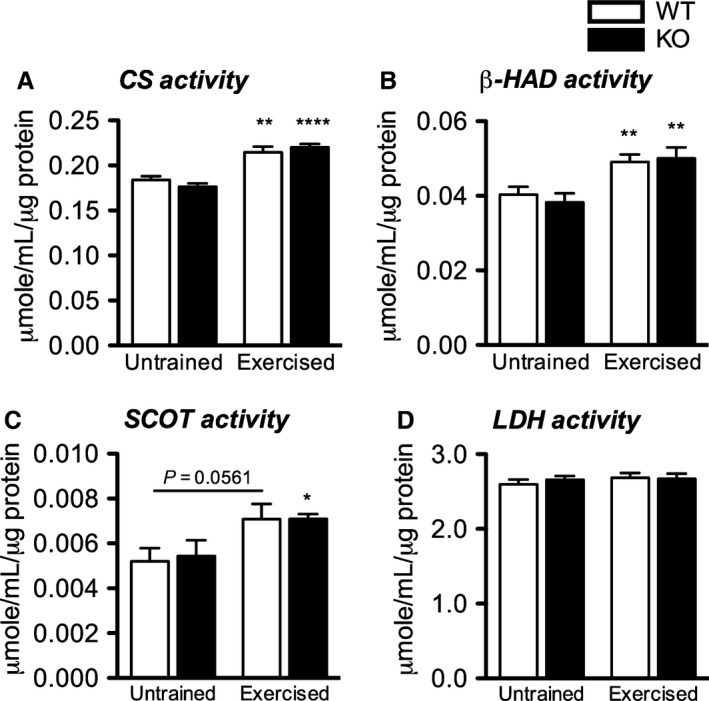
Training‐induced improvements in muscle mitochondrial enzyme activities involved in substrate oxidation are not dependent on CD36. (A) CS, (B) *β*‐HAD, (C) SCOT and (D) LDH activity after 30 day of treadmill exercise in the muscle of WT and CD36 KO mice. Data are expressed as mean ± SEM (*n* = 7–9). No interaction effect was observed as assessed by two‐way ANOVA. Differences between training status of the same genotype were assessed by Student's unpaired *t*‐test where single, double and quadruple *represent *P* < 0.05, 0.01 and 0.0001, respectively.

In the liver, CS activity was not influenced by training nor the absence of CD36 (not shown). In the muscle, however, activities of mitochondrial enzymes in the citric acid cycle (CS; *P* < 0.0001–01), fatty acid *β*‐oxidation (*β*‐HAD; *P* < 0.01) and ketone body oxidation (SCOT; *P* = 0.0561 and *P* < 0.05) were all increased by exercise training (Fig. 6A–C) independent of CD36. The activity of LDH, a cytosolic enzyme participating in anaerobic glycolysis, was neither influenced by training nor CD36 (Fig. 6D).

**Figure 7:**
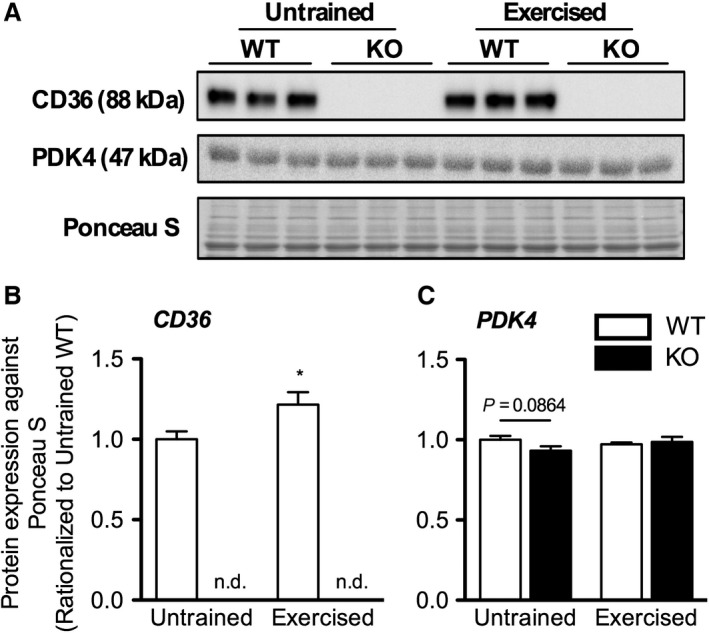
Protein expression of CD36 and PDK4 in WT and CD36 KO mice after training. (A) Representative immunoblot images of CD36, PDK4 and digitized image of Ponceau S stained membrane. Quantification of signals corresponding to (B) CD36 and (C) PDK4 bands rationalized to Ponceau S signal. Values are presented as mean ± SEM (*n* = 7–9) of data from three separate membranes. Membranes were processed in parallel and all groups were represented in each membrane. No significant interaction effects were observed as assessed by two‐way ANOVA. Significant difference (*P < 0.05) was observed in CD36 expression between exercise status of WT mice as assessed by Student's unpaired *t*‐test. Signal corresponding to CD36 was not observed in CD36 KO mice.

Training led to a significant increase (21%; *P* < 0.05) in total CD36 protein expression in the gastrocnemius of WE (Fig. 7A,B). As expected, no signal was detected in K. On the other hand, training did not significantly influence the PDK4 content in the gastrocnemius (Fig. 7A,C).

## Discussion

The properties of exercised skeletal muscle on fatty acid uptake, oxidation and mitochondrial respiration in CD36 KO muscle have been characterized in an ex‐vivo experimental system (McFarlan et al. 2012). However, the contribution in endurance of circulating fatty acids, glucose homeostatic systems and other factors external to the muscle could not be accounted for in this type of set‐up. In this study, we measured whole‐body metabolism at rest and during exercise, exogenous glucose oxidation and changes in metabolites in exercise, gene expression in the muscle, and enzyme activities after training to supplement previous observations. Furthermore, we demonstrate that CD36 is essential not only for basal endurance but also for its training‐induced improvement in whole animals.

Exercise training adaptations in mitochondrial machinery (e.g., increase in electron transport chain and oxidative proteins, and enzyme activity) are associated with improvements in endurance (Holloszy and Coyle 1984; Cartoni et al. 2005; Rangwala et al. 2010). However, the balance between fatty acid supply and catabolism in the mitochondria appears to be more important during exercise as demonstrated in mice lacking HSL, ATGL, and CD36 but having intact mitochondrial function (Fernandez et al. 2008; Huijsman et al. 2009; McFarlan et al. 2012; Fujitani et al. 2014). Above the observed impaired endurance in the untrained state, we show the absence of its amelioration with training despite unaltered adaptations in mitochondrial density and function (Larsen et al. 2012) in CD36 KO mice. This is in stark contrast to improved endurance in WT mice. Exercise capacity as measured by maximal oxygen consumption, and oxygen consumption at submaximal exercise intensity are similar between genotypes (McFarlan et al. 2012). Because we did not measure maximal oxygen consumption, whether our training protocol improved exercise capacity in WT and CD36 KO mice remains to be determined. However, we show that changes in metabolite handling as an adaptation to training contributed to the observed improved endurance in WT mice during our exhaustion‐to‐exercise test as discussed in the succeeding sections.

Changes in the skeletal muscle with training can manifest in whole‐body metabolism both at rest and during exercise. At rest, training induced a photophasic shift in RER and an overall increase in oxygen consumption in WT mice. While circadian effects on activity were not observed, our data suggests the involvement of CD36 in the effects of training on the peripheral clocks (Yasumoto et al. 2015), and whole‐body metabolic activity without accompanying muscle PDK4 protein increase. During exercise, CD36 KO mice had elevated RER and exogenous glucose oxidation indicating carbohydrate as the preferred substrate (McFarlan et al. 2012; Fujitani et al. 2014). We show that training caused a decrease in RER associated with reduced exogenous glucose utilization without changes in oxygen consumption in WT mice suggesting the occurrence of substrate switch to fat during exercise and this adaptation is impaired in the absence of CD36. As glucose handling nor *Glut4* expression were unchanged with training, the contribution of elevated fatty acid uptake with increased CD36 protein and its sarcolemmal localization with contraction could contribute to substrate selection (Rennie et al. 1976; Jeppesen et al. 2011). These data demonstrate the influence of CD36‐mediated fatty acid uptake on the control of mitochondrial substrate selection and oxidation (Holloway et al. 2009; McFarlan et al. 2012) and show that this phenomenon is observable in whole animals both at rest and during exercise. To compensate for decreased intramuscular lipid energy substrates in trained CD36 KO mice, elevated glycogenolysis, and anaerobic glycolysis likely occurred as suggested by lower oxygen consumption, increased muscle lactate and glycogen depletion (Rogatzki et al. 2015). Accumulated lactate might be locally oxidized because blood lactate was not increased basally and during exercise (Rogatzki et al. 2015).

Training increases muscle glycogen and this improves endurance by retarding the depletion of circulating glucose and hepatic glycogen (Baldwin et al. 1973; Pederson et al. 2005; Manabe et al. 2013). The absence of CD36 did not impair nor improve skeletal muscle glycogen accumulation with training. Conversely, the observed elevated muscle glycogen in untrained CD36 KO mice might be caused by promotion of glycogenesis with decreased fatty acid availability (Cazzolli et al. 2002). In the case of liver glycogen, the diet used in the study and 48 h recovery time may not be sufficient to fully recover hepatic glycogen as diets high in fat delay its replenishment after exercise (Conlee et al. 1990; Taylor et al. 2006) in trained WT mice. In relation to this, constant elevated circulating NEFA in CD36 KO mice may cause low basal hepatic glycogen. However, the level of glycogen is not a determinant of endurance per se as its utilization is influenced by the sparing effect of fatty acids (Rennie et al. 1976; Hickson et al. 1977). Indeed, the elevation of serum NEFA and uptake through CD36 during exercise, and elevated basal muscle glycogen in conjunction with suppressed exogenous glucose oxidation resulted in hepatic glycogen sparing in trained WT mice during exercise which likely influenced endurance improvement despite a relatively lower basal hepatic glycogen than its untrained counterpart. In CD36 KO mice, on the other hand, unaltered high demand for glucose without improvement in fatty acid uptake and substrate switch despite increased muscle glycogen led to early onset of hypoglycemia and fatigue (Nybo 2003; Newsholme and Blomstrand 2006). Overall, our data demonstrate that training‐induced muscle glycogen adaptation is intact in CD36 KO mice but this insufficient to ameliorate exercise intolerance and substrate selection during exercise. This also suggests that the role muscle glycogen in endurance is auxiliary to fatty acid uptake and oxidation.

Molecular adaptations related to lipid handling and mitochondrial biogenesis induced by training in the muscle involves the PPARs (*α* and *β*/*δ*) and ERRs (*α* and *β*/*γ*) in transcriptional activation cascade through the co‐activator PGC1*α* (Muoio et al. 2002; Tanaka et al. 2003; Schreiber et al. 2004; Rangwala et al. 2010; Fan and Evans 2015). Decreased fatty acid availability in CD36 KO muscle during a single bout of exercise and through recovery likely impaired effective upregulation of *Pgc1a* via decreased fatty acid activation of PPAR*β*/*δ* (Hondares et al. 2007; Mottillo et al. 2012) despite having increased basal *Ppard* (Drover and Abumrad 2005) and not because of impaired AMPK activation as it is not different between WT and CD36 KO after exercise (McFarlan et al. 2012). In chronic exercise however, adaptations involving calcium handling with increased motor activity (Olson and Williams 2000) could lead to the autoregulatory feed‐forward loop of PGC1*α* participated by calcineurinA, calcium/cadmodulin‐dependent protein kinase IV (CaMKIV) and myocyte enhancer factor 2 (MEF2) (Handschin et al. 2003) and potentially other transcription factors (Schreiber et al. 2004; Jäger et al. 2007; Kleiner et al. 2009) which may explain the difference in expression after a single bout and chronic exercise. On the other hand, *Ppara* but not *Ppard* was increased in the WT with training. ERR*α*, an early PGC1*α* response gene that is upregulated with exercise, controls the transcription of PPAR*α* (Huss et al. 2004; Mootha et al. 2004; Cartoni et al. 2005). Increased fatty acid uptake with elevated CD36 protein expression may upregulate *Ppara* in trained WT muscle as PPAR*α* positively regulates its own transcription (Pineda Torra et al. 2002). In CD36 KO muscle, it appears that elevated basal *Ppara* and *Ppard* are likely controlled by other mechanisms (Drover and Abumrad 2005).

As PGC1*α* is increased, PPARs, ERRs, and other transcription factors are activated and downstream targets are induced. ERRs particularly ERR*β*/*γ* are constitutively active and binding of PGC1*α* potentiates its activity (Greschik et al. 2002; Willy et al. 2004). Indeed, ERR‐dependent mitochondrial biogenesis‐related gene expression (*Pgc1a*,* Cycs* and *Oxct1*) and enzyme function were not dependent on CD36 as previously reported (Rangwala et al. 2010; McFarlan et al. 2012; Svensson et al. 2016). However, unlike the ERRs, PPARs require fatty acids or other lipid ligands for transcriptional activity (Kliewer et al. 1997; Armstrong et al. 2014). Coinciding with increased CD36 protein and *Ppara* mRNA expression, PPAR targets such as fat utilization genes (*Cd36*,* Fatp1*,* Lpl*) were significantly increased (Ehrenborg and Krook 2009; Kleiner et al. 2009) with training in WT but not in CD36 KO muscle. In brown adipocytes and cardiac muscle, cell types that show genetic similarity with skeletal muscle, fatty acid availability through increased lipolysis is required for maximal induction of PPAR*α* and PPAR*β*/*δ* controlled oxidative genes (Haemmerle et al. 2011; Mottillo et al. 2012). Therefore, it is likely that the absence of CD36, that is to say impaired fatty acid availability, prevented significant increases in gene transcription of fat utilization genes through the PPAR pathway.

Intriguingly, apparent but nonsignificant increase in transcription of some PPAR target genes (e.g. *Lpl*,* Fatp1*,* Myh2*,* Vegfa*, and *Mct1*) (König et al. 2008; Ehrenborg and Krook 2009; Kleiner et al. 2009; Gan et al. 2013) was also observed in trained CD36 KO muscle. Assuming corresponding increase in protein expression, this suggests that elevated basal *Ppara*,* Fatp1*, and training‐induced *Pgc1a* in CD36 KO muscle (possibly other fatty acid transporters as well) could induce the same PPAR targets but not with similar efficiency as afforded in the presence of CD36 (Hostetler et al. 2009; Nickerson et al. 2009). Likewise, transcriptional overlap or cooperativity of PPARs and ERRs could not be discounted (Huss et al. 2004; Wang et al. 2004; Rangwala et al. 2010). While contribution of some fatty acid transporters to PPAR signaling have been demonstrated in previous studies [e.g., FABP5 to PPAR*δ* (Armstrong et al. 2014), FABP1 to PPAR*α* (Hostetler et al. 2009) and FABP4 to PPAR*γ* (Tan et al. 2002)], their independent and cooperative function require further investigation.

In summary, we report that CD36 is necessary for basal endurance and its improvement or amelioration with training independent of elevation in mitochondrial function, and muscle glycogen accumulation. In addition, we show that training effects on substrate switch at rest and during exercise is impaired in the absence of CD36. These changes, at least in WT mice, possibly involve CD36 in efficient upregulation of exercise‐responsive genes controlled by PPARs.

Humans deficient in CD36 have decreased aerobic exercise capacity because of limited fatty acid uptake in muscle and heart (Yanai et al. 2007; Hames et al. 2014). Because training could not improve endurance performance in the absence of CD36, frequent glucose supplementation may benefit human athletes with CD36 deficiency during prolonged exercise bouts.

## Conflict of Interest

None declared.
